# Immunotherapy progress and clinical strategy of unresectable locally advanced non-small cell lung cancer

**DOI:** 10.3389/fonc.2023.1022042

**Published:** 2023-02-08

**Authors:** Xiaofei Zhang, Jianguo Zhang, Peiyi Liu, Juan Wang, Kuaile Zhao, Zhengfei Zhu, Kangsheng Gu, Weixin Zhao

**Affiliations:** ^1^ Department of Radiation Oncology, Fudan University Shanghai Cancer Center, Shanghai, China; ^2^ Department of Oncology, Shanghai Medical College, Fudan University, Shanghai, China; ^3^ Department of Oncology, Lu‘an civily Hospital, Anhui, Lu’an, China; ^4^ Department of Orthopedics, TongRen Hospital, School of Medicine Shanghai Jiao Tong University, Shanghai, China; ^5^ Department of Oncology, The Second People’s Hospital of Kashgar, Kashgar, China; ^6^ Department of Oncology, The First Affiliated Hospital of Anhui Medical University, Hefei, China

**Keywords:** unresectable locally advanced NSCLC, immunotherapy, radiotherapy, immunotherapy progress, clinical strategy

## Abstract

Non-small cell lung cancer negative for actionable molecular markers entered the splendid era of immunotherapy. This review aims to provide an evidence-based summary for immunotherapy for unresectable locally advanced non-small cell lung cancer, and references for clinical strategies of immunotherapy. Through literature review, the standard treatment for unresectable locally advanced non-small cell lung cancer should be radical concurrent radiotherapy and chemotherapy followed by consolidation immunotherapy. However, the efficacy of concurrent radiotherapy, chemotherapy combined with immunotherapy has not been improved, and its safety should be further validated. It is believed that induction immunotherapy plus concurrent radiotherapy and chemotherapy plus consolidation immunotherapy is promising. In clinical practice, the delineation of radiotherapy target should be relatively small. Pemetrexed combined with PD-1 inhibitor induces the strongest immunogenicity in chemotherapy, which is suggested by preclinical pathway study. Although there is no significant difference between PD1 and PD1 for effect, PD-L1 inhibitor is better in the combination treatment of radiotherapy which presents significantly less adverse events.

## 1 Background

At present, stage IV non-sensitive mutations in non-small cell lung cancer (NSCLC) which means negative for actionable molecular markers of EGFR mutation, ALK,ROS1, BRAF, NTRK1/2/3, METex14 skipping, RET has entered the splendid era of immunotherapy, while its application to unresectable locally advanced NSCLC is still being explored. This review aims to provide an evidence-based summary for immunotherapy for unresectable locally advanced NSCLC, and references for clinical strategies of immunotherapy.

## 2 Previous therapeutic principles for unresectable locally advanced NSCLC

### 2.1 Definition of locally advanced NSCLC

According to the AJCC Cancer Staging Manual, 8^th^ Edition, stage III NSCLC is categorized into stage IIIA (T3/T4N1) and stage IIIB (T3/T4N2, T1/T2N3 and T3/T4N3) (**Table 1**). Furthermore, it is classified into surgery-treated stage III (mainly stage IIIA) and radiotherapy-treated stage III (mainly stage IIIB/IIIC). In the present study, we aim to review the treatment of unresectable locally advanced NSCLC.

**Table 1:** NSCLC staging in AJCC Cancer Staging Manual, 8th Edition.

	N0	N1	N2	N3
T1a	IA1	IIB	IIIA	IIIB
T1b	IA2	IIB	IIIA	IIIB
T1ac	IA3	IIB	IIIA	IIIB
T2a	IB	IIB	IIIA	IIIB
T2b	IIA	IIB	IIIA	IIIB
T3	IIB	IIIA	IIIB	IIIC
T4	IIIA	IIIA	IIIB	IIIC
M1a	IVA	IVA	IVA	IVA
M1b	IVA	IVA	IVA	IVA
M1c	IVB	IVB	IVB	IVB

### 2.2 A Therapeutic strategy: Concurrent or sequential radio-chemotherapy?

Radical dose of radiotherapy alone was the main therapeutic strategy for unresectable locally advanced NSCLC before 1990. Three classic studies in the 1990s showed that the radiochemical comprehensive therapeutic strategy is better than that of radiotherapy alone (1–3). Therefore, it is believed that the comprehensive radiotherapy and chemotherapy achieves a better efficacy than that of radiotherapy alone.

Later, great efforts have been made on exploring the preference of concurrent or sequential radiotherapy and chemotherapy. A randomized phase III trial (RTOG 9410) in 2011 involving 577 patients compared the efficacy of concurrent or sequential strategy (4). It is shown that the progression free survival (PFS, 14.6 months vs. 17.0 months, *P*<0.05) and the 5-year overall survival (OS, 10% vs. 16%, *P*<0.05) are worse in patients receiving sequential radiotherapy and chemotherapy than those treated with the concurrent strategy. A meta-analysis involving 1,205 patients with unresectable stage III cancer in 6 large-scale clinical trials demonstrated that patients gain more benefits of 3-year and 5-year OS from concurrent radical radiotherapy and chemotherapy than those treated with the sequential strategy (5). Notably, the incidence of grade 3-4 acute esophageal adverse event is significantly higher in the concurrent group than that of sequential group (18% vs. 4%, *P*=0.001). It is concluded that concurrent radiotherapy and chemotherapy is preferred if cancer patients can be well tolerant.

### 2.3 The higher radiotherapy dose, the better therapeutic efficacy?

RTOG 0617 analyzed the efficacy of 60 Gy or 74 Gy radiotherapy with concurrent paclitaxel plus carboplatin chemotherapy with or without cetuximab in the era of three-dimensional radiotherapy (6). It is shown that the survival is better in 60 Gy group than that of 74 Gy group, which is mainly attributed poor prognosis to higher adverse events.

In recent years, other researches on improving the radiotherapy efficacy by enhancing the dose is on ongoing. In the RTOG 0617 clinical trial, the radiotherapy dose increases to 74 Gy for the entire irradiated target area. Such a simple and direct dosage increase method needs to be optimized. Novel dosage increases methods include imaging-guided local increase, dose and fractionation radiotherapy, and individualized adaptive radiotherapy under the tolerant range. A study based on functional image-guided adaptive radiotherapy found that individualized adaptive radiotherapy after determination of the target by FDG-PET is an acceptable strategy to control the local cancer in unresectable stage II-III NSCLC patients (7). In the present study, the radiotherapy dose adaptively increases based on metabolism change detected by FDG PET-CT, and then dose and fractionation are accelerated based on the tolerance of normal tissues. Clinical data of RTOG1106 were reported at the IASLC 2020 World Conference on Lung Cancer in Singapore(WCLC), suggesting the great safety and efficacy of the individualized adaptive radiotherapy. However, whether it can benefit from PFS and OS remains to be further verified (8). Taken together, the higher dose of concurrent radiotherapy and chemotherapy is not better by now.

### 2.4 Selection of chemotherapy in the concurrent radiotherapy and chemotherapy

CAMS study is a head-to-head comparative RCT (Randomized Controlled Trial) of concurrent radiotherapy and chemotherapy of EP (etoposide and cisplatin) and PC (paclitaxel and carboplatin) for local stage III NSCLC (9). Compared with those of EP group, the 3-year OS (41.1% vs. 26%, *P*=0.024) and the median OS (23.3 months vs. 20.7 months, *P*=0.094) in PC group were significantly lower. Therefore, based on 2017 CAMS study the standard regimen is still EP.

Another classical clinical trial of PROCLAIM compared pemetrexed plus cisplatin and EP in the concurrent radiotherapy at 66 Gy/33f and chemotherapy in locally advanced nonsquamous NSCLC (10). No significant differences in the 3-year and 5-year OS are detected between groups, although the PFS is prolonged in the pemetrexed plus cisplatin group. In addition, the incidence of grade 3-4 drug-related adverse events is significantly lower in the pemetrexed plus cisplatin group than that of EP group, including neutropenia. Subgroup analyses further demonstrated that stage IIIB and large planning target volume(PTV, > 700 mL) are independent factors for the poor prognosis. Notably, subgroup patients gain more benefits of OS from the administration of pemetrexed, which is recommended to NSCLC patients with the large tumor volume or advanced stage.

One phase II noncomparative randomized trial was conducted to determine the optimal sequencing and integration of paclitaxel/carboplatin with standard daily thoracic radiation therapy. Patients with unresected stages IIIA and IIIB NSCLC received two cycles of induction paclitaxel (200 mg/m2)/carboplatin followed by radiotherapy 63.0 Gy (arm 1, sequential) or two cycles of induction paclitaxel (200 mg/m2)/carboplatin (AUC = 6) followed by weekly paclitaxel (45 mg/m2)/carboplatin (AUC = 2) with concurrent radiotherapy 63.0 Gy (arm 2, induction/concurrent), or weekly paclitaxel (45 mg/m2)/carboplatin (AUC = 2)/radiotherapy (63.0 Gy) followed by two cycles of paclitaxel (200 mg/m2)/carboplatin (AUC = 6; arm 3, concurrent/consolidation). As a result, median overall survival was 13.0, 12.7, and 16.3 months for arms 1, 2, and 3, respectively. Taken together, Concurrent chemotherapy regimens for all histologic primary treatments include cisplatin/etoposide and carboplatin/paclitaxel (11). For NSCLC, additional concurrent chemotherapy regimens can be used, including carboplatin/pemetrexed and cisplatin/pemetrexed.

### 2.5 Concurrent radiotherapy and targeted therapy

Relevant studies on the concurrent targeted therapy are relatively limited. The Chinese clinical trial of RECEL involving 41 unresectable stage III NSCLC patients combined with EGFR mutations in exon 19 or 21 compared PFS as the primary outcome between erlotinib group (n=20) and EP group (n=21) (12). The PFS is significantly longer in erlotinib group than that of EP group (24.5 months vs. 9.0 months, HR(Hazard Ratio) = 0.104, *P*<0.001). Besides, objective response rate (ORR) in EP group and erlotinib group is 70% and 61.9%, respectively. The incidence of all-grade adverse event is similar between groups (88.9% vs. 84.2%). However, this trial is limited by the small sample size, and more clinical data are needed to support the clinical strategy of concurrent radiotherapy and targeted therapy for NSCLC.

### 2.6 Is maintenance chemotherapy necessary?

The KCSG-LU05-04 is a stage III RCT analyzing consolidation chemotherapy with or without docetaxel and cisplatin after concurrent radiotherapy and chemotherapy (13). A total of 437 patients were recruited, and they were intervened with 20 mg/m^2^ docetaxel (1×6cy qw) plus 20 mg/m^2^ cisplatin (1×6cy qw) and 66 Gy/33 f radiotherapy. After the whole course, patients are assigned into observation group and consolidation group, respectively for analyzing the primary outcome of PFS. Although PFS is slightly longer in consolidation group than that of observation group (9.1 months vs. 8.1 months), no significant difference is detected (*P*=0.36). OS is comparable between groups as well. Therefore, consolidation chemotherapy is not recommended after concurrent radiotherapy and chemotherapy. The cycle of concurrent radiotherapy and chemotherapy is two cycles.

The previous treatment strategies for unresectable locally advanced NSCLC are summarized as follows: 1. Concurrent radiotherapy and chemotherapy serves as the standard radical treatment, which is better than that of sequential treatment. 2. Radical radiotherapy at 60 Gy was preferred than that at 74 Gy. 3. EP is the standard chemotherapy regimen of concurrent radiotherapy and chemotherapy. However, pemetrexed plus platinum can be used for non-squamous NSCLC with poor general condition and large volume) 4. Clinical data of concurrent radiotherapy and targeted therapy are limited compared with that of standard concurrent radiotherapy and chemotherapy, which requires further explorations.

## 3 Immunotherapy for unresectable locally advanced NSCLC

### 3.1 Summary of consolidation immunotherapy

#### 3.1.1 PACIFIC trial

Prior to the application of immunotherapy, the median OS in patients with unresectable locally advanced NSCLC after the standard treatment was reported as 14.5 months which is not ideal. The PACIFIC trial in 2017 reported encouraging data that created the new era of immunotherapy (14). It was a randomized, double-blind phase III trial evaluating 713 patients with unresectable stage III NSCLC. After at least two cycles of standard concurrent radiotherapy and cisplatin-based chemotherapy, those without disease progression are further subjected to the randomization for 42 days. In a 2:1 ratio, eligible patients are assigned into durvalumab(PDL1) consolidation group and placebo group, and those in the former are intervened with 10 mg/kg durvalumab q2W for 1 year. PFS and OS are the primary endpoints. In 2020, the 4-year OS and PFS are reported by annual meeting of European Society for Medical Oncology(ESMO) The median OS (47.5 months vs. 29.1 months), 4-year OS (49.6% vs. 36.3%), median PFS (17.2 months vs. 5.6 months) and 4-year PFS (35.3% vs. 19.5%) are significantly higher in consolidation group than those of placebo group. Subgroup analysis showed a relative decline in the incidence of new lesions in all cases, especially in the brain, where the incidence descended nearly doubled. The safety is acceptable regardless of all-grade or grade 3-4 adverse events. In addition, pneumonia as the adverse event has been well concerned. The incidence of pneumonia (33.9% vs. 24.8%) and grade 3-4 adverse event (3.4% vs. 2.6%) are comparable between consolidation group and placebo group, suggesting the good safety and tolerance.

However, the subgroup analysis showed that the therapeutic efficacy is not ideal in patients with positive mutations of EGFR. It is suggested that the initial EGFR gene status should be detected in all patients, and those with positive mutations of EGFR should no longer performed durvalumab consolidation therapy. *Post hoc* analysis of PD-L1 expression showed that Patients with PD-L1 < 1% may gain clinical benefits of PFS, rather than OS from durvalumab consolidation treatment. Therefore, durvalumab consolidation treatment should be carefully considered in patients with low expression levels of PD-L1 (< 1%).

Memorial Sloan Kettering Cancer Center (MSKCC) conducted a PACIFIC study related failure mode analysis analyzed the failure pattern involving 62 cases with at least one course of durvalumab consolidation treatment were retrospectively analyzed (15). The local recurrence and distant metastasis are 18% and 30%, respectively. In addition, the rate of oligometastasis ranks 47% in the distant metastasis group (n=18), who were theoretically have the opportunity to receive radical radiotherapy of SBRT(Stereotactic Body Radiation Therapy).

In addition, another analysis of the failure patterns of researchers in the PACIFIC study was reported at the 2019 ASCO meeting (16) The overall progression risk in durvalumab consolidation group is reduced compared with that of placebo group (45.4% vs. 64.6%). Furthermore, the intrathoracic progression risk (36.6% vs. 48.1%) and the incidence of intrathoracic progression (80.1% vs. 74.5%) which suggests that the rate of intrathoracic recurrence is still relatively high in the treated group and the local therapeutic efficacy should be further enhanced. About 66.6% of patients developed 1-2 oligometastases in the extrathoracic progression group, suggesting that these patients may benefit from SBRT to control the metastasis.

#### 3.1.2 Clinical trial of LUN 14-179

LUN14-179 is phase II study with single arm of patients with unresectable stage III NSCLC received concurrent chemoradiation with cisplatin and etoposide, cisplatin and pemetrexed, or carboplatin and paclitaxel and 59.4 to 66.6 Gy of radiation (17). Patients with nonprogression of disease were enrolled and received pembrolizumab (200 mg intravenously every 3 weeks for up to 12 months). PFS reported in the LUN 14-179, durvalumab consolidation group of PACIFIC trial and placebo group of PACIFIC trial is 18.7, 17.2 and 5.6 months, respectively, and OS is 35.8, 47.5 and 29.1 months, respectively. It is indicated that similar to the findings in the PACIFIC trial, consolidation treatment involving immune drugs achieves more clinical benefits than that of placebo group (**Table 2**).

**Table 2:** Efficacy results of consolidation immunotherapy for LUN 14-179 and PACIFIC trial.

Endpiont	LUN 14-179	PACIFIC(Durvalumab)	PACIFIC(Placebo)
PFS*	18.7	17.2	5.6
12m-PFS**	60.80%	55.70%	35.30%
18m-PFS	46.90%	49.50%	27.00%
OS*	35.8m	47.5m	29.1m
12m-OS	81.30%	83.10%	74.60%

*The value shows the median and the unit is month.

**m represents month.

#### 3.1.3 Consolidation dual-immunotherapy

Consolidation treatment of PD-1 or PD-L1inhibitors significantly enhances OS in patients with unresectable locally advanced NSCLC following concurrent radiotherapy and chemotherapy. However, the safety and efficacy of dual-immunotherapy of CTLA-4 and PD-1 inhibitors are unclear. In the LUN 16-081 trial reported by ASCO in 2020, a total of 105 cases of unresectable stage IIIA/IIIB NSCLC patients receiving chemotherapy are recruited in this randomized, multi-center phase II clinical trial. They are randomly assigned into nivolumab(PD1) group and nivolumab/ipilumab(CTLA-4 inhibitor) group in a 1:1 ratio. 480 mg nivolumab iv q4w and nivolumab 3mg/kg iv q2w plus ipilumab 1mg/kg iv q6w are given for 24 weeks in the two groups, respectively. The safety is analyzed in 50 patients, and the data showed that the incidence of adverse events increases in nivolumab/ipilumab group than that of nivolumab group. Results from nivolumab alone group were consistent with previous studies of other immunotherapy drugs. The above data provide a evidence-based medical evidence that the nivolumab consolidation treatment after concurrent radiotherapy and chemotherapy may be promising. An interim safety analysis of 50 patients showed a higher incidence of adverse events in the nivolumab/ipilumab group than in the nivolumab group (18). With subsequent data updates, 18-month PFS were 62.3% vs. 67% for both groups, median PFS were 25.8 vs. 25.4 months, and estimated 18-month and 24-month OS rates were 82.1% and 76.6% vs. 85.5% and 82.8%, respectively (unpublished data at 2022 ASCO#8509). These data provide a new idea for consolidation therapy of nivolumab after concurrent chemoradiotherapy. Dual immunotherapy combined with consolidation therapy is not recommended due to safety and efficacy.

The CONSIST trial conducted by Professor Yu Jinming in China is ongoing for analyzing consolidation immunotherapy (Unpublished data, NCT03884192). It is a phase III RCT analyzing sintilimab(PD1) consolidation after concurrent radiotherapy and chemotherapy in unresectable locally advanced NSCLC patients.

Another phase III clinical trial of Gemstone-301 analyzed patients with unresectable stage III NSCLC who do not have disease progression after concurrent/sequential radiotherapy and chemotherapy, which is the only study for self-produced anti-PD-L1 inhibitor in China. CS1001 1200 mg q3w and placebo are given for 24 months, followed by observation of the primary outcome (PFS). The clinical data of GEMSTONE-301 were orally reported at ESMO in 2021 (19). A pre-planned interim analysis conducted after a median follow-up of 14 months showed that the median PFS assessed by independent review committee (BICR)in the CS1001 group and the placebo group is 9.0 months and 5.8 months, respectively, with an HR of 0.64. The 12-month PFS is 45% and 26%, respectively, and the 18-month PFS is 39% and 23%, respectively. Experimental procedures in the GEMSTONE-301 have been modified based on the PACIFIC trial (14), which not only recruits unresectable stage III NSCLC patients with more actual conditions for sequential radiotherapy and chemotherapy, but also excludes patients with EGFR/ALK/ROS1 mutations who are highly detected in East Asian populations and not well responsive to immunotherapy. As a result, the GEMSTONE-301 trial is more accordance to the real-world experience with less heterogeneity of subjects on therapeutic efficacy.

### 3.2 Concurrent radiotherapy, chemotherapy and immunotherapy

Based on the above-mentioned analyses, consolidation immunotherapy therapy after concurrent radiotherapy and chemotherapy have become the new standard for the treatment of locally advanced NSCLC. Can the curative effect be improved by further advancing immunotherapy to the stage of concurrent radiotherapy and chemotherapy? The following research provides some evidence.

#### 3.2.1 DETERRED trial

DETERRED trial is a phase II study involving 2 parts (20). During the part 1, patients are intervened by concurrent radiotherapy and chemotherapy, followed by atezolizumab(PDL1) consolidation treatment for 2 cycles for 1 year (n=10). Part2 is to advance atezolizumab to the concurrent radiotherapy and chemotherapy stage, and then combine with atezolizumab to consolidation chemotherapy 2cycles, atezolizumab maintained for 1 year (n=30). The median PFS (18.6 months vs. 13.2 months) and OS (22.8 months vs. not achieved) in part 1 and 2 are calculated. Compared with the PACIFIC trial (14), PFS is similar in part 1, but OS is worse in part 1. Part 2 advanced atezolizumab to concurrent radiotherapy and chemotherapy, and PFS did not improve as compared to Part One or PACIFIC studies. The incidence of immune-related adverse events above grade 3 (30% vs. 20%) and pneumonia above grade 2 (10% vs. 16%) in part 1 and part 2 are comparable, suggesting the acceptable safety. However, it is limited by a small sample size (n=40). Definite evidences supporting the priority of immunotherapy before concurrent radiotherapy and chemotherapy are lacked.

#### 3.2.2 NICOLAS trial

NICOLAS is a single-arm phase II trial for analyzing the efficacy and safety of nivolumab combined with concurrent radiotherapy and chemotherapy in unresectable locally advanced stage III NSCLC (21). A total of 79 patients treated with nivolumab 360mg q3w and concurrent radiotherapy and chemotherapy with chemo of Cisplatin plus Etoposide/Pemetrexed/Vinorelbine, and Carboplatin plus (etoposide, pemetrexed, vinorelbine), followed by nivolumab 480mg consolidation treatment for 1 year were recruited. Grade ≥3 pneumonitis (CTCAE v4.0) up to 6 months post-radiotherapy is measured as the primary outcome. A total of 165 radiotherapy-related adverse events occurred, including 22 grade 3, 3 grade 4, and 1 grade 5 adverse events (bronchiopulmonary hemorrhage, esophageal fistula). There are 240 immune-related adverse events, including 26 grade 3, 5 grade 4 and 4 grade 5 adverse events (colitis, pulmonary fibrosis, autoimmune disease, pneumonia). Generally, there are 77 patients receiving at least one trial, regardless of treatment related or not, including 34 patients with pneumonia (7 of grade 3 and 1 of grade 5), 24 with esophagitis (5 of grade 3), and 27 with dyspnea (2 of grade 3). The incidence of overall adverse events is high. In addition, 6/79 patients develop grade 5 toxicity. The median PFS and OS are 12.7 months, and 38.8 months (updated), respectively, which are higher than the median PFS (9.8 months) reported in the PROCLAIM trial. However, survival data are not exciting compared with the median PFS (17.2 months) and OS (47.5 months) reported in the PACIFIC trial. Concerning the safety and efficacy, it is not recommended to additionally perform immunotherapy during the concurrent radiotherapy and chemotherapy.

### 3.3 Induction immunotherapy plus concurrent radiotherapy and chemotherapy plus maintenance immunotherapy

Based on the above studies, simultaneous chemoradiotherapy plus immunotherapy is not advocated. It is speculated whether the immunotherapy can be first performed as the induction treatment. The AFT-16 trial conducted in ASCO Mayo Clinic in 2020 involving 64 subjects in 13 medical centers analyzed induction immunotherapy of atezolizumab (22). A total of 62 patients are treated with at least one time of atezolizumab intervention. After 12-week induction, the disease control rate (DCR) achieves 77.4%, which is 75.8% after 6-week induction. In this trial, 49 subjects are subjected to the detection of PD-L1. DCR in the PD-L1<1% group and PD-L1>1% group is 82.4%, and 90.9%, respectively. Therefore, the curative effect is expected. Adverse events are reported in 54 cases, and most of which are grade 1, including hyperthyroidism, hypothyroidism, skin rash, allergies, colitis, and Guillain-Barré syndrome. It is suggested that the safety of induction immunotherapy of atezolizumab is acceptable. Therefore, the mode of induced immunotherapy plus concurrent radiotherapy and chemotherapy followed by maintenance immunotherapy is promising, and we look forward to its final results.

**Figure 1:**
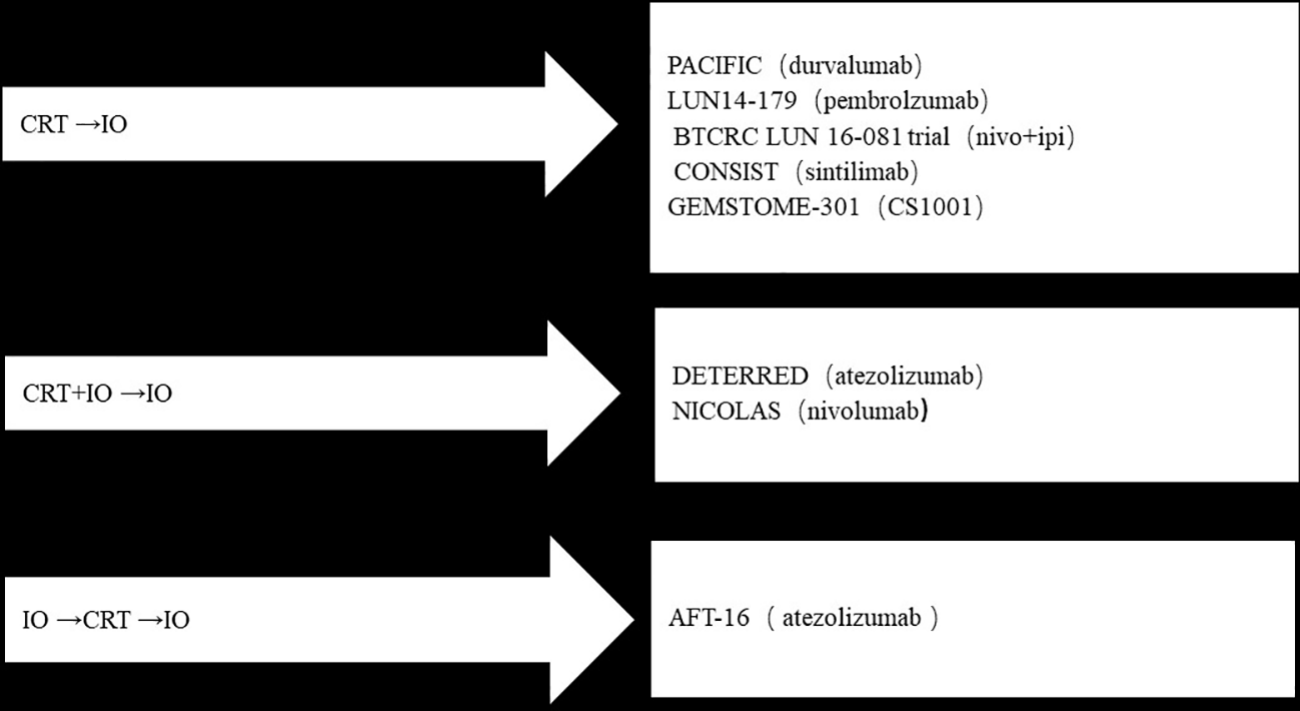
Summary of all the immunotherapy studies of locally advanced NSCLC.

The above is our summary of all the immunotherapy studies in the era of locally advanced NSCLC (**Figure 1**). The conclusion is that the standard treatment for unresectable locally advanced NSCLC is radical radiotherapy and chemotherapy followed by consolidation immunotherapy, The efficacy of concurrent chemoradiotherapy and concurrent immunotherapy has not been significantly improved, and its safety needs to be further verified. Induction immunotherapy, followed by concurrent radiotherapy and chemotherapy and maintenance immunotherapy is promising, and relevant clinical data are expected to be reported in the future (**Table 3**). 

**Table 3:** Study summary.

Trial Name	Trial number	Stages		Patient number	Treatment regimens	Adverse events	mPFS (m)	mOS (m)
**RTOG 0617 (****6**) ***	NCT 00533949	III	Arm1	217	CRT(60Gy)/+ Cetuxi	Above grade3 (76%)	11.8	28.7
			Arm2	207	CRT(74Gy)/+ Cetuxi	Above grade3 (79%)	9.8	20.3
			Arm3	237	Cetuxi+CRT(60Gy/74 Gy)	Above grade3 (86%)	10.8	25
			Arm4	228	CRT(60Gy/74Gy)	Above grade3 (70%)	10.7	24
**CAMS (****9****)**	NCT 01494558	III	Arm1	95	CRT(EP)	Grade ≥3 esophagitis(20%)	14.0	23.3
			Arm2	96	CRT(PC)	Grade ≥3 esophagitis(6.3%)	12.0	20.7
**PROCLAIM (****10****)**		III	Arm1	283	Pem-Cis-TRT	Grade3/4(64%)	11.4	26.8
			Arm2	272	Eto-Cis-TRT	Grade3/4(76.8%)	9.8	25.0
**RECEL (****12****)**	NCT 01714908	II	Arm1	20	RT+Erlotinib	Any grade(88.9%)	24.5	–
			Arm2	21	RT+EP	Any grade(84.2%)	9.0	–
**KCSG-LU05-04 (****13****)**	NCT 00326378	III	Arm1	211	CRT		8.1	20.6
			Arm2	209	CRT>DP		9.1	21.8
PACIFIC (14)	NCT02125461	III	Arm1	476	CRT*>Durva	Grade3/4(3.4%)	17.2	47.5
			Arm2	237	CRT>Placebo	Grade3/4(2.6%)	5.6	29.1
LUN 14-179 (17)	NCT02343952	II	–	92	CRT>Pembro	Above grade3 (6.5%)	18.7	35.8
LUN16-08 (18)	NCT03285321	II	Arm1	52	CRT> Nivo	Grade3/4(32%)	25.8	–
			Arm2	47	CRT> Nivo+Ipilu	Grade3/4(44%)	25.4	–
Gemstone-301 (19)	NCT03728556	III	Arm1	255	RT>sugemalimab	Grade 3/4(9%)	9.0	–
			Arm2	126	RT>Placebo	Grade 3/4(6%)	5.8	–
DETERRED (20)	NCT03102242	II	Arm1	10	CRT>Atezo	Above grade3 (30%)	18.6	22.8
			Arm2	30	CIRT*(Atezo)	Above grade3 (20%)	13.2	Not achieved
NICOLAS (21)	NCT02434081	II	–	79	CIRT*(Nivo)>Nivo	Severe AEs(11.6%)	12.7	38.8
AFT-16 (22)	NCT03102242	II	–	62	Atezo>CRT> Atezo	Grade3/4(4.8%)	–	–
RTOG 9311 (23)	NCT 00002577	II	–	177	–	–	–	–
CONSIST^**^	NCT03884192	III	Arm1	–	CRT-Sinti	–	–	–
			Arm2	–	CRT-Placebo	–	–	–

**Unpublished. *CRT, Concurrent Chemoradiotherapy; CIRT, Concurrent Chemoradiotherapy and immunotherapy; >, Sequential therapy; TRT, Thoracic Radiation Therapy.

***The superior number represents the number of references.

## 4 Thinking about the clinical strategy of unresectable locally NSCLC in the immunotherapy era

### 4.1 Improvement of radiotherapy targets and techniques in the immunotherapy era

Involved-field irradiation (IFI) was recommended mainly based on the following studies. RTOG 9311 analyzed 179 patients with local and regional treatment failure after radiotherapy who are followed up for 16 months (23). Among 31 cases of local recurrence, 28 cases have a clear record of the recurrence site, and only 14 (9%) have recurrences outside the radiation field, Therefore, it is suggested that irradiation involving field is sufficient. Another trial published in 2007 involved 200 patients with stage III NSCLC (24). Patients are randomly grouped into IFI group and elective nodal irradiation (ENI) group, and they are intervened by 2 cycles of chemotherapy plus radiotherapy using the three-dimensional radiotherapy technology. It is shown that the incidence of radiation-induced lung injury (17% vs. 29%) and local failure rate (41% vs. 49%, *P*<0.044) are lower in IFI group than those of ENI group, suggesting the comparable efficacy but higher toxicity in ENI group. Therefore, IFI is adopted in the targeted area of radiotherapy and recommended by the NCCN guidelines.

A study published in 2014 analyzed lymphocytes of the immune system in 711 patients who underwent radical radiotherapy (25). It is found that gross tumor volume (GTV)is correlated with lymphopenia. The increase in the volume of the target area increases with lymphopenia. Regardless of combined chemotherapy or not, the degree of lymphocyte decline is correlated with OS and PFS, that is, the more severe lymphocytes decrease, the worse the prognosis. In addition, the lung V5, which refers to the volume percentage of the whole lung exposed to radiation dose of 5 Gray or more, is mostly correlated with the decline of lymphocyte count, with the maximum R(correlation coefficient) between V5 and exposure dose. Compared with photon irradiation, the use of proton technology has a better effect on lymphocyte decline.

Compared with photon radiotherapy, the main advantage of particle radiotherapy is its precise dose distribution ability. The Prague peak phenomenon brings unique dose distribution characteristics, which allows particles to be highly integrated and delivered to tumors at high doses, and avoids damage to surrounding normal tissues. These characteristics make particle radiotherapy become a treatment option for LA-NSCLC, which can avoid damaging the heart, spinal cord, esophagus and other important organs near the lesion, and is superior to photon therapy in reducing the dose of key structures in the chest (26). In addition, compared with photon radiotherapy, particle radiotherapy has greater potential to induce immune death, especially heavy ion radiotherapy (27).

Therefore, in the immune era, the target area should be relatively small. IFI irradiation technology can protect the immune function of normal lymphatic tissues. On the other hand, radiotherapy technology needs to transform from two-dimensional to three-dimensional or four-dimensional, so it can reduce the movement of the target to reduce the irradiation range of the target. In the selection of rays, if possible, high-energy rays such as protons or heavy ions can protect normal tissues better than X-rays.

### 4.2 Selection of chemotherapy in the concurrent radiotherapy and chemotherapy in the era of maintenance immunotherapy

One *in-vivo* study in 2019, MC38 and Colon26 cells that are sensitive to both pemetrexed and PD-L1 inhibitors are selected for analyses (26). Flow cytometry data showed an increase in immune cells after 50 mg/kg pemetrexed induction. QuantiGene gene expression analysis of MC38 tumors showed that the increase in factors related to immune activation is more obvious in 50 mg/kg pemetrexed group than that of 100 mg/kg pemetrexed group. In addition, compared with paclitaxel group, carboplatin group, pemetrexed plus carboplatin group and paclitaxel plus carboplatin group, pemetrexed can induce more immune-activating cytokines. In terms of tumor growth, pemetrexed combined with PD-L1 inhibitor group had the slowest tumor growth rate compared with pemetrexed alone and PD-L1 group. According to the prognosis curve, the combination group showed a better prognosis. Correlation analysis revealed that pemetrexed plus PD-L1 inhibitor group produced the highest number of immune factors, followed by the PD-L1 monotherapy group. Therefore, pemetrexed combined with immune drugs presents the strongest anti-tumor effect and yields the best prognosis, suggesting the synergistic effect of pemetrexed on immune drugs. Another study reported that pemetrexed (cisplatin is not contained) combined with PD-1/PD-L1 can inhibit tumor growth by activating and/or recruiting tumor-infiltrating CD4+ and CD8+ T lymphocytes, which provides references for the regulatory mechanisms of pemetrexed in improving the immune checkpoint block (ICB) of lung cancer (28). It is believed that pemetrexed combined with immunotherapy is a preferred choice. Another basic study showed that pemetrexed or gemcitabine can effectively induce the expression of PD-L1 and sensitizes the immune system. However, cisplatin, paclitaxel and vinorelbine cannot effectively induce its expression (29).

There are also hints in practical clinical studies. In the clinical trail of KEYNOTE-799, patients with unresectable locally advanced stage III NSCLC who are intervened by pembrolizumab combined with concurrent radiotherapy and platinum-containing dual-drug chemotherapy for the first-line treatment are recruited (30). They are initially categorized into cohort A (squamous NSCLC) and cohort B (nonsquamous NSCLC). Paclitaxel plus carboplatin plus pembrolizumab for 1 cycle, followed by pembrolizumab plus paclitaxel plus carboplatin conbine chest radiotherapy are performed in the former cohort. After the treatment, pembrolizumab consolidation immunotherapy for a total of 17 cycles is given. Patients in cohort B are treated with 1-week induction therapy of pemetrexed plus cisplatin plus pembrolizumab, followed by pembrolizumab plus pemetrexed combined chest radiotherapy. After the treatment, pembrolizumab consolidation immunotherapy for a total of 17 cycles is given. The overall treatment pattern is immunochemotherapy induction plus triple concurrent treatment plus consolidation immunotherapy. ORR and the incidence of grade 3 pneumonia are the primary outcomes and PFS, OS and safety are the secondary outcomes to be measured. The first 15-week follow-up results (published in 2020 ASCO) showed that the incidence of ≥ grade 3 pneumonia in cohort A and B is 8% and 5.5%, respectively. The ORR is 67.0% and 56.6%, respectively, and that of sustained remission for more than 6 months is 91.1% and 100%, respectively. The 6-month PFS (81.4% vs. 85.2%) and 6-month OS (87.2% vs. 94.8%) are both lower in cohort A than those of cohort B. After follow-up for 6 months, ORR in cohort A reaches 70.5%. The 1-year OS in both cohort A and B is higher than 80% (81.3% vs. 87%). It is suggested that pemetrexed combined with immunotherapy maybe a good choice for nonsquamous NSCLC patients.

### 4.3 Selection of immune drugs

**Figure 2:**
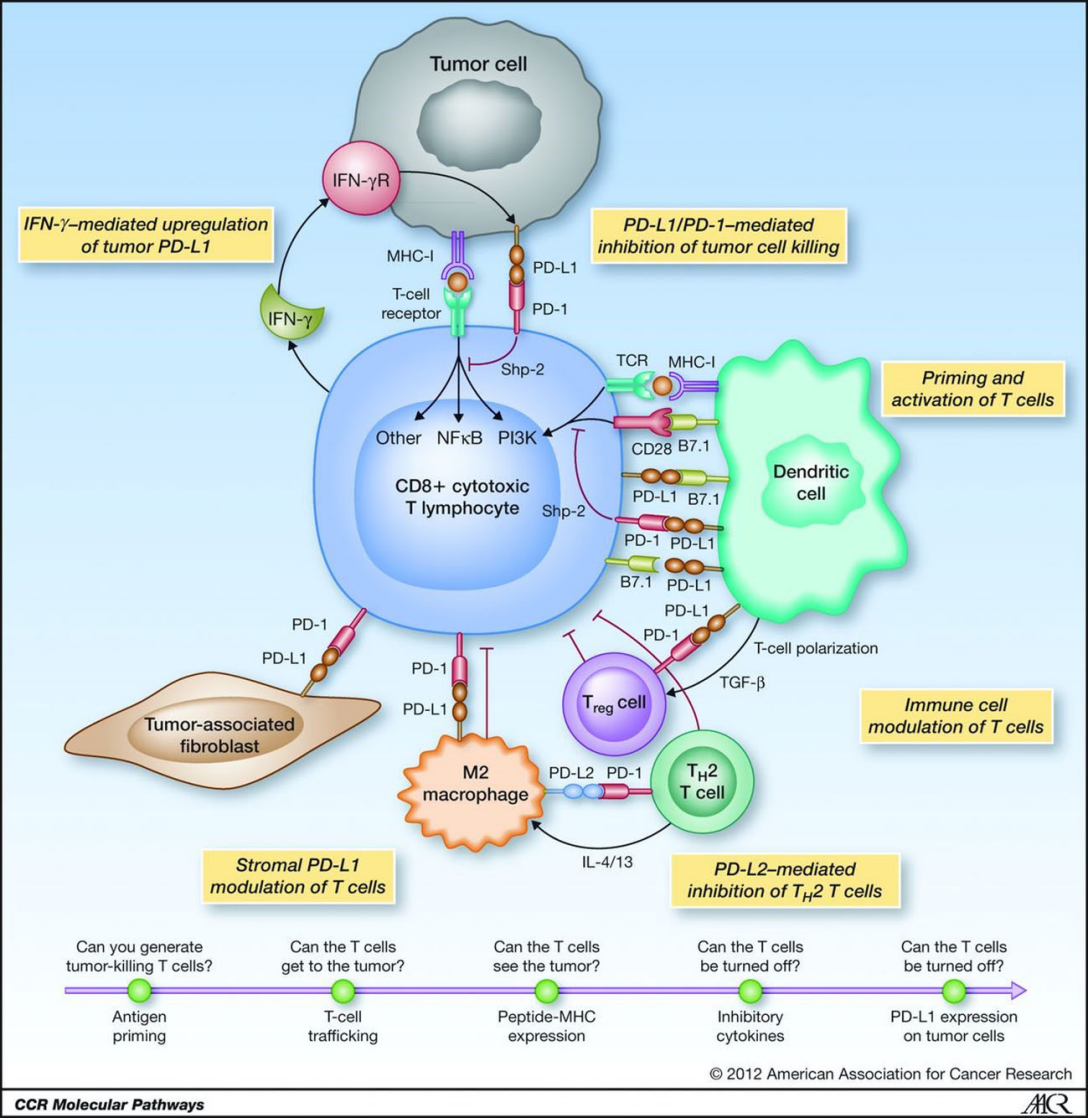
Tumor immunology and the PD-L1/PD-1 pathway.

It is well known the PD-L1 pathway downregulates the immune response, which can be upregulated by blocking it. PD-L1 is distributed on tumor cells and macrophages, and PD-1 is distributed on activated T cells. PD-1 inhibitors not only block PD-L1, but also PD-L2. Blocking PD-L2 upregulates the host’s immune response and increase the occurrence of autoreactive inflammation. However, PD-L1 inhibitors only block the function of PD-L1, but retains that of PD-L2 on macrophages, thus maintaining the autoimmune homeostasis *via* avoiding the occurrence of autoreactive inflammation. As

a result, safety of PD-L1 inhibitor is theoretically better than PD-L inhibitor (31) (**Figure 2**). Through literature review of immunotherapy of PD-1 and PD-L1 inhibitors (14), adverse event of pneumonia is well concerned, which is one of the most frequent adverse events in immunotherapies. It is shown that the incidence of interstitial pneumonia after PD-L1 inhibitor treatment is also lower than that of PD-1 inhibitor (32).

The comparative study on the efficacy of PD-L1 inhibitors and PD-1 inhibitors for local advanced NSCLC is limited, but the study of efficacy of immunotherapy for stage IV NSCLC showed similarity for PD-L1 inhibitors and PD-1 inhibitors (33–37). Based on the safety and efficacy, PD-L1 inhibitors is superior to PD-1 inhibitors in the occurrence of adverse events, and PD-L1 inhibitors is preferred in the case of equivalent efficacy. When related adverse reactions occur, timely detection of patients’ symptoms and timely use of glucocorticoids are the most important.

## 5 Conclusion

At present, the standard treatment for unresectable locally advanced NSCLC should be radical radiotherapy and chemotherapy followed by consolidation immunotherapy. However, the efficacy of concurrent radiotherapy and chemotherapy combined with immunotherapy has not been improved, and its safety should be further validated. Through literature review, we believed that induction immunotherapy plus concurrent radiotherapy and chemotherapy plus consolidation immunotherapy is promising. In clinical practice, the delineation of radiotherapy target should be relatively small and selective lymph node irradiation is not recommended. three-dimensional or four-dimensional radiotherapy radiotherapy or proton/heavy ion therapy is the optimal radiotherapy technique. Compared with paclitaxel and carboplatin, pemetrexed combined with PD-1 inhibitor induces the strongest immunogenicity in chemotherapy (38). In addition, PD-L1 inhibitor is better than PD-1 inhibitors in the combination treatment of radiotherapy, which presents significantly less adverse events.

## Author contributions

XZ, KG substantial contributions to the conception and design of the study, acquisition of the data and analysis and interpretation of the data. PL, JZ, WZ draft the article or revising it critically for important intellectual content. ZZ and JW approved the final version to be published. All authors contributed to the article and approved the submitted version.
